# Species ethnobotanical values rather than regional species pool determine plant diversity in agroforestry systems

**DOI:** 10.1038/s41598-021-03408-3

**Published:** 2021-12-14

**Authors:** Daniel K. N’Woueni, Orou G. Gaoue

**Affiliations:** 1 Faculty of Agronomy, University of Parakou, 01BP 123, Parakou, Benin; 2 Department of Ecology and Evolutionary Biology, University of Tennessee, Knoxville, TN 37996 USA; 3 Department of Geography, Environmental Management and Energy Studies, University of Johannesburg, APK Campus, Johannesburg, South Africa

**Keywords:** Agroecology, Biodiversity, Forestry, Tropical ecology

## Abstract

The conversion of natural systems into farms and agroecosystems is the main cause of biodiversity loss. In human-dominated landscapes, understanding the interactions between agroforestry systems and adjacent natural vegetation is fundamental to developing sustainable agricultural systems. Species can move between these two systems with natural systems providing the regional pool of species that shape the agricultural values and conservation value of the agroforestry systems. We investigated the influence of neighboring natural habitats on traditional agroforestry systems in the buffer zone of Pendjari Biosphere Reserve in Benin to understand the contribution of regional processes on the quality of agroforestry systems. We expected that agroforestry parklands adjacent to natural vegetation with high species diversity will also have higher plant species diversity. We found no similarity in plant species composition between agroforestry systems and adjacent natural habitats. A small proportion of species in adjacent natural habitats were found in agroforestry systems. The proportion of shared species was not significantly influenced by plant diversity in adjacent natural habitats or the distance from the agroforestry systems to the natural adjacent habitat. However, plant diversity in agroforestry systems was strongly associated with site ethnobotanical values indicating that farmers act as a supplemental but severe environmental filter of the regional species pool. Our study suggests that promoting the plantation of plants with high ethnobotanical use-value is a potentially viable strategy for sustainable agriculture and ecological restoration in Biosphere reserves.

## Introduction

Understanding how multiple factors govern species diversity in ecosystems and the practical consequences for species diversity is a core question in ecology and conservation biology^1,2^. The spatial patterns of species diversity is driven by a wide range of ecological processes including facilitation^3^, competition^4^, disturbance^5–7^, productivity^6,8^ and productivity–disturbance interactions^9^. Communities tend to be highly diversified when ecological processes provide more favorable conditions for species coexistence^9–11^. The environmental filtering hypothesis suggests that local species assemblages are the result of the filtering of regional species pools by constraints posed by abiotic and biotic factors^12–14^. Empirical evidence of environmental filtering is rare^15^. Local species assemblages may result from the single or interactive effects of several drivers including landscape or ecosystem modifications by human activities^16,17^.

In human modified landscapes, understanding the interactions between agricultural systems and adjacent natural habitats is fundamental to developing sustainable agricultural systems^18,19^. Environmental filtering can shape diversity in agricultural systems with consequences on the composition of plants that can migrate from nearby natural habitat to act as weed to crops. In addition, plant diversity in adjacent natural habitat can shape arthropod community diversity^20–22^ which can affect crop herbivory rate and ultimately crop yield^23^. The strength of environmental filtering in shaping species assemblages in agroforestry systems will directly depends on the dispersal abilities of species from the regional pools^24–27^ but also on the distance from these pools. Consistent with the island biogeography theory^28,29^, the closer the agroforestry systems are to the regional species pools, the more likely are species going to disperse into agroforestry systems and the higher diversity and shared species richness are expected.

Biodiversity conservation in biosphere reserves is increasingly centered around the effort to minimize the impact of chronic anthropogenic disturbance given the persistent encroachment of croplands into these protected areas^30^. Biosphere reserves are structured into three zones with different functions^31^: (i) a core zone, which is strictly protected and where only research and ecological monitoring activities are allowed, (ii) a buffer zone used for low impact tourism, forestry, and agriculture in line with overall conservation objectives and (iii) the transition zone, often the largest, where a variety of human activities are allowed^32,33^. In the transition zone of most African biosphere reserves, there is an increasing transformation of the natural vegetation into islands of wilderness surrounded by farms and agroforestry systems. This is due to the rapidly increasing world population, land shortage in rural areas and pressure from agricultural intensification^34,35^. Investigating the factors that shape diversity in the traditional agroforestry systems in the transition zone of Biosphere reserves is central to maintaining the double role of protected areas to safeguarding long-term ecological sustainability and satisfying basic human needs^36^.

**Figure 1:**
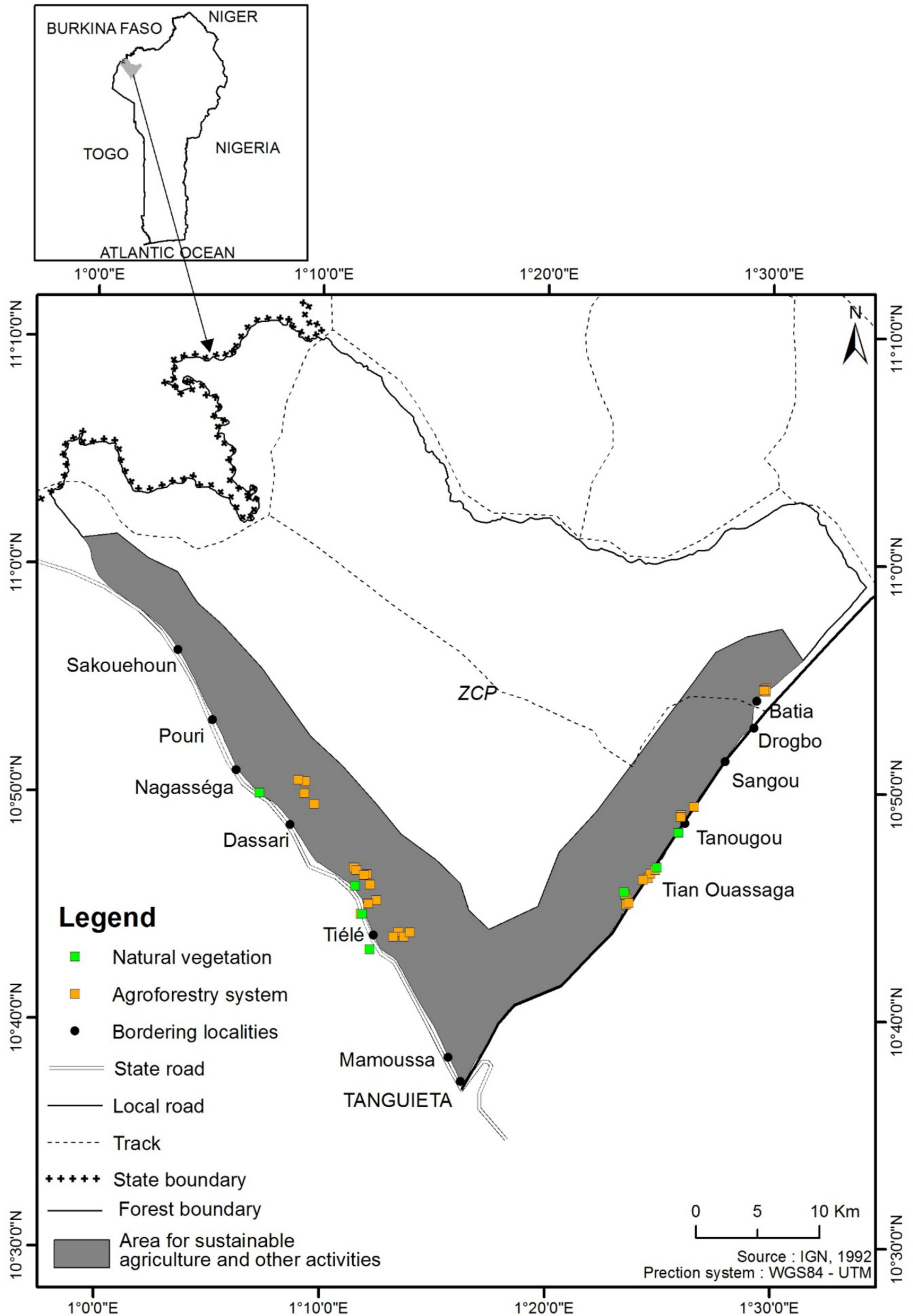
Map of the hunting zone of the Pendjari Biosphere Reserve in Northern Benin showing study sites in agroforestry parklands that are established in the transition zone where sustainable agricultural activities are allowed. The transition zone is composed of two narrow strips of land on each side of the hunting zone (grey). On the Tanguieta-Batia road axis, populations are constrained by the Atacora Chain of Mountains which boarded the eastern side of the hunting zone limiting agricultural expansion. The map was created using ArcGIS 10.5.1 (ESRI, https://www.esri.com).

We used the theoretical framework of the environmental filtering and island biogeography theories to examine the effects of various socio-cultural and agricultural (ethnobotanical value, agricultural practices) and proximity to natural habitat (distance from adjacent natural vegetation) on species diversity in traditional agroforestry systems in the Pendjari biosphere reserve in Benin (west Africa, Fig. 1;^37^). We analyzed the changes in species diversity at different levels of agricultural land use intensity. Consistent with the island biogeography theory, we hypothesized that the distance from agroforestry systems to adjacent species pools of natural vegetation will predict the number of species in agroforestry systems and the proportion of shared species between the two plant communities. The closer the agroforestry systems are to their surrounding natural vegetation, the greater number of plant species the two communities will share and the higher the species richness will be in the agroforestry systems. However, we hypothesized that the regional species pool will be filtered by abiotic/biotic factors but also the influence of local farmers who decide what species to clear from or add to their farms based on their ethnobotanical value. We hypothesized that species ethnobotanical values along with the proximity of agroforestry to natural vegetation will predict local species assemblages in agroforestry parklands.

## Results

### Similarity in diversity and species composition between agroforestry systems and adjacent natural vegetation

**Figure 2:**
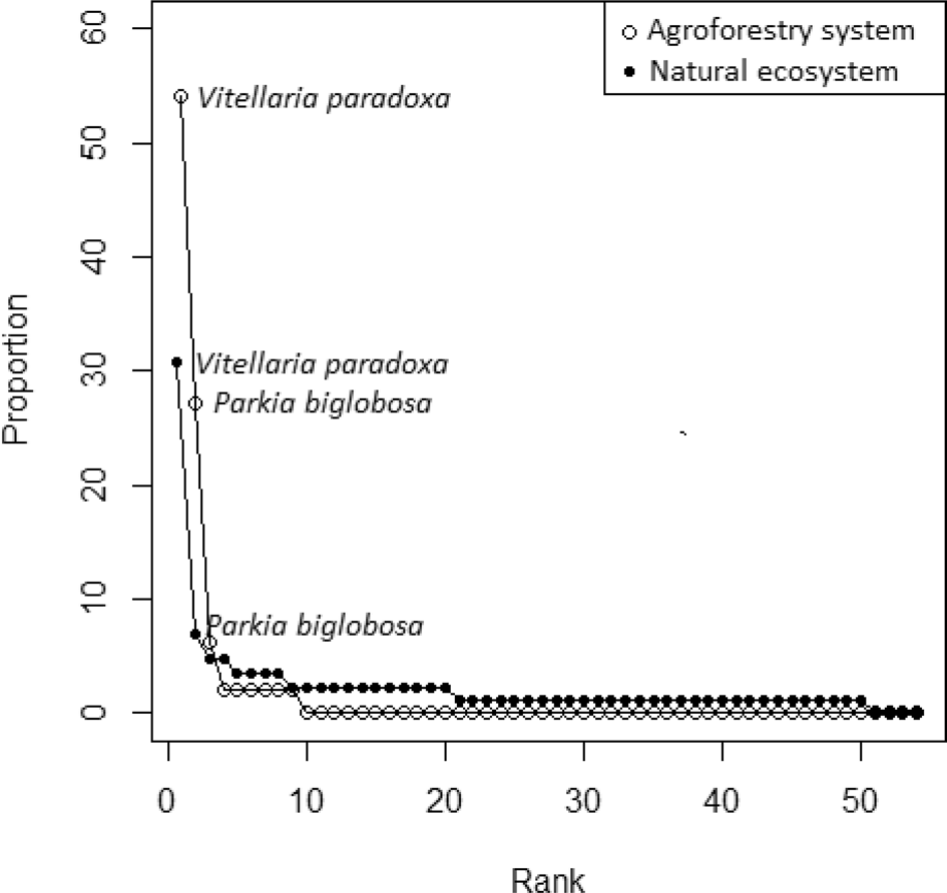
Rank-frequency diagrams of adjacent natural formations and traditional agroforestry systems of Pendjari Biosphere Reserve.

The rank-frequency diagram for the agroforestry systems was different from that of the adjacent natural habitat (Fig. 2). We estimated a total of 8 species in the agroforestry systems against 31 species in adjacent natural habitats indicating a very low diversity in agroforestry systems. In addition, individuals were more fairly distributed among species in the adjacent natural habitat than in the agroforestry systems. However, we found similarities in relative abundance of a few species. For example, *Vitellaria paradoxa* was the most dominant species in both ecosystems followed by *Parkia biglobosa*. However, species composition besides these dominant species were different between the two ecosystems. For example, in addition to *V. paradoxa* and *P. biglobosa*, the agroforestry systems were represented by only singleton species (5 species) whereas adjacent natural habitat included singleton (12 species) and doubleton (7 species). In addition, regular species found in the adjacent natural habitat included *Combretum fragans, Terminalia avicennioides, Burkea africana* whereas only *Lannea acida* was represented in agroforestry systems.

**Figure 3:**
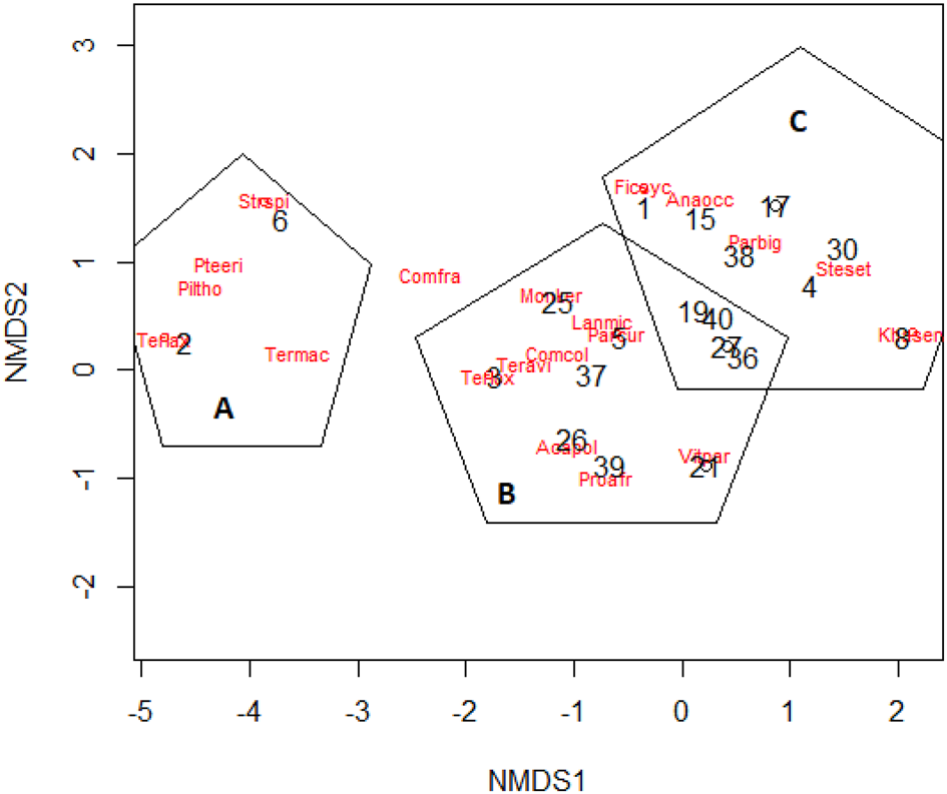
Nonmetric dimensional scaling analysis showing the dissimilarity between agroforestry systems and their surrounding natural vegetation. NMDS identified three plant communities (**A**–**C**). Numbers represent plots from adjacent natural vegetation (1–8) or from agroforestry systems (8–40). The words in red represent the plant species. Termac = *Terminalia macroptera*, Pteeri = *Pterocarpus erinaceus*, Piltho = *Piliostigma thonningii*, Teravi = *Terminalia avicennioides*, Comfra = *Combretum fragran*s, Vitpar = *Vitellaria paradoxa*, Proafr = *Prosopis africana*, Anaocc = *Anarcadium occidentale*, Parbig = *Parkia biglobosa*, Steset = *Sterculia setigera*, Comcol = *Combretum collinum*, Monker = *Monotes kerstingii*, Acapol = *Acacia polyacantha*, Lanmic = *Lannea microptera*.

The non-metric multidimensional scaling (NMDS) identified three groups of plant communities (Fig. 3). The first group (A) was composed of two plots that were all established in the natural habitats especially in savannah and rocky outcrop habitat. This first group was highly dissimilar to the others two groups which included plots from moist savannah with unique species such as *Pterocarpus erinaceus, Piliostigma thonningii, Terminalia macroptera, Terminalia avicennioides*. These two plant communities (B and C) were more similar and included a mixture of agroforestry systems plots (plot #1, 3, 4,5, 7, and 8) and those of adjacent natural vegetation. Typical agroforestry species such as *Parkia biglobosa, Vitellaria paradoxa* and *Anacardium occidentale* were shared between these two communities.

### The influence of regional species pools on agroforestry species assemblages

**Figure 4:**
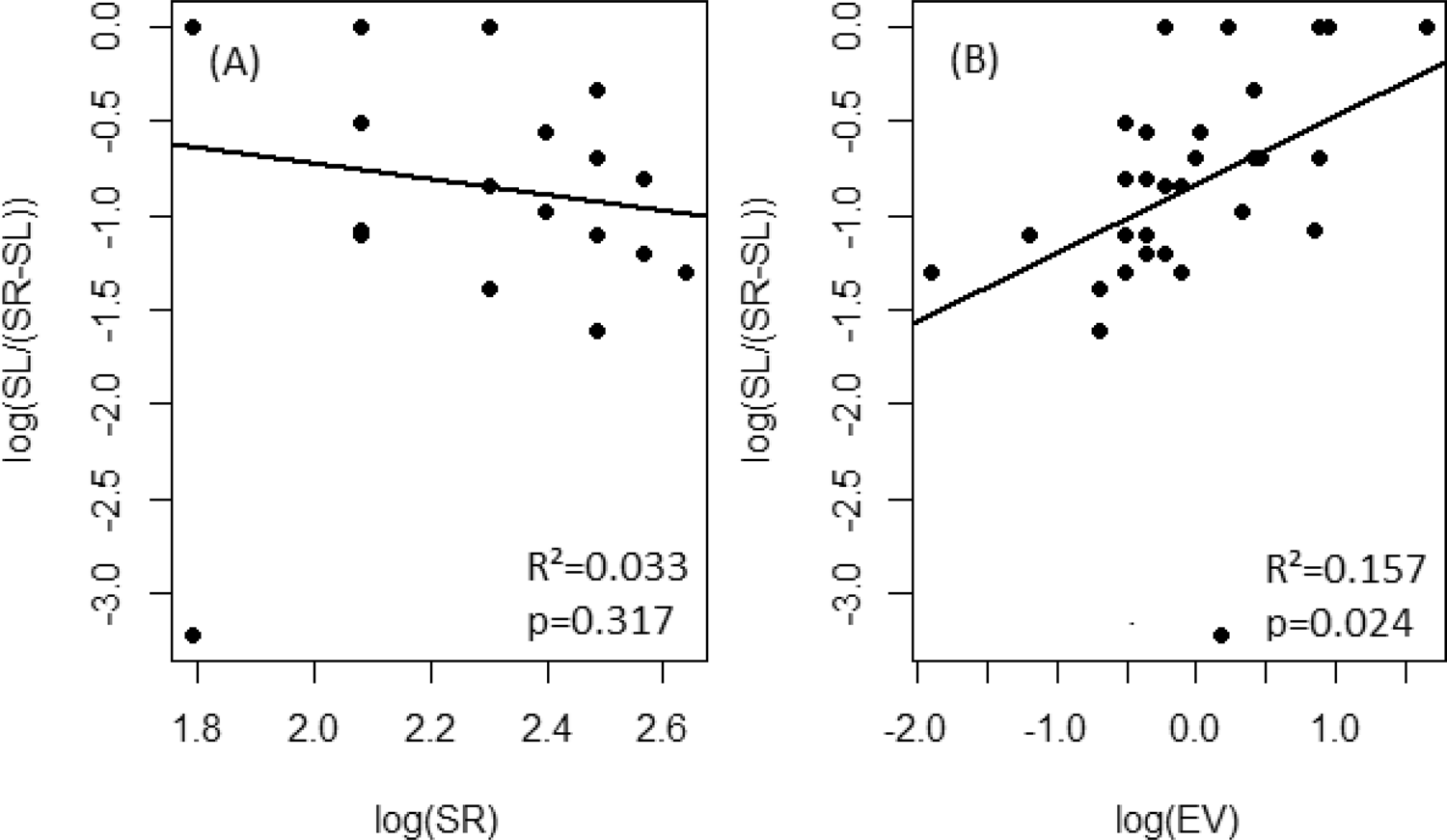
Log–log regression illustrating (**A**) the non-significant linear decrease in the proportion of shared richness as function of regional species pool (SR) and (**B**) the linear increase in the proportion of shared richness as function of ethnobotany value (EV). SL is the species richness of agroforestry system.

We found no significant relationship between the agroforestry system and adjacent natural habitat for species richness (β = 0.138 ± 0.259, *P* = 0.910) and Shannon diversity (β = 0.542 ± 0.348, *P* = 0.370; Table 1). However, the proportion of species richness shared between agroforestry systems and their adjacent natural habitats was positively affected by the ethnobotanical values (β = 0.362 ± 0.152; *P* = 0.024, R^2^ = 0.15, Fig. 4B, Table 2) but was not significantly associated with the regional species pool (β = − 0.426 ± 0.419; *P* = 0.318, R^2^ = 0.033, Fig. 4A).

**Table 1:** Bootstrap regression testing the linear relationships of species richness and Shannon diversity index between agroforestry systems and adjacent natural vegetation.

	Estimate	*P* value	BootBias	BootSE	BootMed	5%	95%
Intercept	1.576	0.380	− 0.103	0.435	1.530	0.963	2.395
Species Richness	0.138	0.910	0.087	0.259	0.153	− 0.374	0.477
Intercept	1.276	0.000	0.000	0.248	1.293	0.850	1.701
Shannon Diversity	0.542	0.370	0.042	0.348	0.542	− 0.078	1.083

**Table 2:** Parameterized models and selection of appropriate models using information theoretic approach.

Models	ΔAIC_c_	df
Distance + ethnobotanical value	0	4
Distance + ethnobotanical value + crop type	1.8	5
Distance + ethnobotanical value + crop type + distance × crop type	1.9	6
Distance + ethnobotanical value + crop type + distance × crop type + ethnobotanical value × crop type	3.5	7
Distance	4.4	3
1	3.6	2

ΔAICc is the small sample corrected difference in Akaike Information Criterion (AIC) between each model and the lowest AIC value. df is the degree of freedom.

### The role of human driven environmental filtering on species assemblages in agroforestry systems

The distance between agroforestry systems and neighboring natural habitats did not significantly affect the proportion of the shared species richness (β = − 0.012 ± 0.199, *P* = 0.950, Table 3). However, plot level species ethnobotanical value positively affected the proportion of the shared species richness (β = 0.701 ± 0.205, *P* = 0.001) indicating that farmers in this case play an important role in filtering species assemblages from the regional pool of the adjacent natural habitat. Furthermore, we found no significant effect of distance on species richness (β = 0.033 ± 0.093, *P* = 0.729, Table 4) and Shannon diversity index (β = − 0.012 ± 0.109, *P* = 0.656, Table 5). Similarly, ethnobotanical value had no significant effect on Shannon diversity index (β = 0.038 ± 0.055, *P* = 0.250) but positively affected species richness (β = 0.173 ± 0.080, *P* = 0.030).

**Table 3:** Beta regression testing the effect of the distance between agroforestry systems and their surrounding vegetation and ethnobotany value on the proportion of shared species.

Variables	Estimate	SE	Z	*P*
Intercept	− 0.524	0.307	− 1.702	0.088
Distance (scaled)	− 0.012	0.199	− 0.062	0.950
Ethnobotanical value	0.701	0.205	3.417	0.001**

Distance was standardized.

**Table 4:** Generalized linear model with negative binomial errors testing the effects of the distance between agroforestry systems and their surrounding vegetation and ethnobotanical value on agroforestry systems species richness.

Variables	Estimate	SE	Z	*P*
Intercept	1.078	0.143	7.545	< 0.0001***
Distance	0.033	0.093	0.359	0.719
Ethnobotanical value	0.173	0.080	2.167	0.030*

**Table 5:** Bootstrap regression testing the effects of the distance between agroforestry systems and their surrounding vegetation and ethnobotanical value on Shannon diversity index.

	Estimate	*P*	BootBias	BootSE	BootMed	5%	95%
Intercept	0.663	< 0.001	− 0.019	0.077	0.653	0.487	0.789
Distance	− 0.012	0.656	− 0.027	0.109	− 0.013	− 0.239	0.029
Ethnobotanical value	0.038	0.250	0.013	0.055	0.035	− 0.015	0.206

## Discussion

This study examined species diversity patterns in agroforestry systems and their surrounding natural vegetation using the theory of environmental filtering and island biogeography theories as theoretical framework. The agroforestry systems around the Pendjari biosphere reserve have particularly low species diversity as compared to the richness of 60 species identified in semi-natural habitats and the 41 species in agroforestry systems in the nearby Vipalogo region of Burkina Faso^38^. This result may be explained by the increasing charcoal production and agricultural intensification within the transition zone that caused the fast decline in some species over time^37^.

Clearly, our rank-frequency analysis showed lower diversity in agroforestry systems than in the natural habitats. Several potential mechanisms can explain such low diversity which may indicate strong environmental filtering of species from the regional species pools. First, the low diversity is perhaps due to the strong selective practice of farmers who prefer to conserve large size trees which provide fodder, fruits as famine food and several other non-timber forest products for local communities. Two main species are spared as a result, including *Vitellaria paradoxa* for its fruits and seeds used for shea butter and seeds of *Parkia biglobosa* used to make traditional mustard^37,39,40^. Small diameter trees, considered as non-desirable for this purpose, are instead harvested for fuelwood directly extirpating these species from the assemblages. Previous studies show that disturbance like fire and grazing can induce small-size trees (sapling or juvenile) mortality^41,42^. However, such effect was not evident in our study system since bush fires are not permitted in the biosphere reserve and grazing in the agroforestry systems take place for limited time and in very few places.

We hypothesized that site ethnobotanical values and proximity to natural habitats are positively associated with species diversity in agroforestry systems. We found support for the first hypothesis that ethnobotanical value was positively correlated with agroforestry systems diversity, but we found no significant correlation with distance. The non-significant effect of distance on species diversity in agroforestry systems is in contrast with predictions form the island biogeography theory. The theory predicts that islands/fragments closer to regional pools (mainland), which are more likely to receive more species dispersed from the regional pool, will have higher species diversity than the one that are farther away^28,29^. However, in our study, the benefit of dispersal in closer agroforestry systems was probably cancelled out by the strong human-driven filtering of species in these agroforestry systems. This is further illustrated by our finding that both agroforestry systems and their adjacent natural habitats were largely dominated by two agroforestry species (*Parkia biglobosa* and *Vitellaria paradoxa*). Farmers tend to conserve these preferred species because of their high nutritional, medicinal and commercial values^43,44^. The versatility of these species which can be used for multiple purposes make them an extremely valuable species to select for in the human modified landscapes^45,46^. Besides, the species filtering around the Biosphere reserve is also driven by agricultural policies that prioritize cash crops over traditional food crops^47,48^ with strong economic and ecological drawbacks^49^.

In addition to the lack of influence of farms proximity to natural vegetation on species assemblages in agroforestry systems, we also found no support for the effect of regional species pool. We hypothesized that if regional species pool had strong influence on agroforestry systems, then species diversity in agroforestry systems will increase with species diversity in neighboring natural habitats. Surprisingly, our results revealed a positive but non-significant correlation for species richness and Shannon index between the two systems, indicating a limited influence of regional species pool on agrobiodiversity. However, the proportion of shared richness was significantly and positively correlated with ethnobotanical values. These results, consistent with our previous analysis, suggest that species of higher ethnobotanical values are more likely to be conserved in agroforestry systems than species of lower ethnobotanical values. This finding highlights the key role of ethnobotanical knowledge in biodiversity conservation^50–52^ and suggests that effort to increase agrobiodiversity must focus on promoting or adding plant species with high ethnobotanical values.

In this study, we investigated the drivers the species diversity patterns in traditional agroforestry systems around an African Biosphere reserve. We found no significant correlation between agroforestry systems and their adjacent vegetation for species richness and Shannon diversity index. We also found no significant effect of proximity to natural vegetation on plant richness in agroforestry systems. However, we found that ethnobotanical value was the key variable that drives the fraction of species diversity shared meaning that while converting natural vegetation into farm, farmers prefer to conserve species of high medicinal, nutritional, and commercial values. This underlines the central role that ethnobotanical knowledge plays in biodiversity conservation. Documenting and using indigenous knowledge on species of high ethnobotanical values in the ecological restoration of agroforestry system could stimulate farmers to conserve more species in their farms than usual. Such commitment on the part of farmers can sustainably improve crop yields (D. K. N’Woueni and O. G. Gaoue, *unpublished data*) and contribute to slowing down deforestation within the Biosphere reserve. We recommend ecological restoration efforts to prioritize species of high ethnobotanical values and particularly versatile plants that are used for multiple purposes.

## Methods

### Study area

The Pendjari Biosphere Reserve covers 4,661.4 km^2^ and is located in the northwest of Benin between 10° 30′ N–11° 30′ N and 0° 50′–2° 00′ E (Fig. 1). The annual rainfall ranges from 1000 to 1100 mm. The rainy season last nearly five months from mid-May to October followed by a dry season from November to February^53^. The vegetation is dominated by woodlands, savannas and grasslands established on poorly developed and tropical ferruginous soils^54^. The Pendjari Biosphere reserve is dominated by open forests and wooded savannas, wooded, shrub and grassy savannas. These plant communities are increasingly degraded by a rapidly growing population^55^.The area is characterized by an annual average temperature of 27 °C. The minima and maxima are respectively of 21 °C and 40 °C^56^. There are rough or little evolved soils, tropical ferruginous soils and hydromorphic soils^54^.

The Pendjari Biosphere Reserve is organized into three main zones: a core area or protected zone (The Pendjari National Park), a buffer zone and a transition zone where sustainable agriculture is permitted^57^. The Pendjari hunting zone is bordered by two main roads (Tanguieta-Batia road, and the Tanguieta-Porga road) which separate it from the transition zone^58^. Along the two main roads and inside the Pendjari hunting zone, local populations establish such a traditional agroforestry systems and gather non-timber forest products within the first 5 km perpendicularly from the roads^59^. Two main ethnic groups are established along the two main roads: the Berba ethnic group which accounts for 65% along the northwestern side and Wama and Gourmantche ethnic groups which account for 30% along the northeastern side^60^. Farms extension in the area occupied by the Wama-Gourmantche is constrained by the Atacora Chain of Mountains. Such a constraint makes the Wama-Gourmantche less environment conservators than the Berba^37,55^.

### Estimating diversity in agroforestry systems and natural vegetation

We conducted a floristic inventory in 32 traditional agroforestry systems and 8 adjacent natural habitats all distributed around 8 villages that border the transition zone of the Biosphere reserve. These villages were selected so that they are closer to the Biosphere reserve and based on the tree density and diversity of agricultural systems. Because the main goal of this study was to understand species assemblages in agroforestry systems, the villages were selected only if they had agroforestry systems that are used by local people. The first set of villages was selected from the Tanguiéta—Batia road and included Tchanwassaga, Pessagou, Tanougou, and Batia (Fig. 1). The second set of villages was selected from the Tanguiéta—Porga road and included Tiélé, Kani, Koupendri, Dassari. The size of the populations in these villages varies from 1169 inhabitants in Tiélé to 7472 inhabitants in Dassari with a mean of 2779 inhabitants per village. Four agroforestry systems were sampled in each village. For a given village, we established one 30 m × 30 m plot in each agroforestry parkland and one plot of the same size was installed in each adjacent natural habitat. The size of our survey plot was chosen based on decades of vegetation surveys in West African savanna which uses plot sizes between 500 and 1000 m^2^^61,62^. In the agroforestry plot, all crop and adult plant with diameter at breast height (DBH) ≥ 5 cm were identified and recorded. In the adjacent natural habitat, we also identified every plant species and estimated their abundance. The distance between the edge of each agroforestry system and the adjacent natural habitat was measured using a Global Positioning System (GPS).

We estimated trees density by counting individual woody plants within each plot. We used this data to develop a matrix of species abundance, each row representing a unique plot and each column representing a species recorded in our survey. We used this matrix to estimate species richness and Shannon diversity index^63^. Shannon index was estimated at the plot level using packages *Hotelling* and *vegan* in R version 3.1.2^64^. We estimated the plot level species richness with Chao abundance-based coverage estimators^65^ using the function “*estimateR*” in package *vegan*. We also used the matrix of species abundance to construct the rank-abundance curves for agroforestry systems and their surrounding natural systems. The proportion of agroforestry systems species richness shared with their adjacent natural habitats was estimated as SL/(SR-SL) with SL the species richness of the agroforestry system and SR the species richness of the adjacent natural plant community.

### Estimating ethnobotanical values

We estimate the total ethnobotanical values of each of the plant species surveyed in the agroforestry systems in the eight villages. We used a snowball sampling method, a sampling approach commonly used in ethnobotany^66–69^ to select and individually interviewed a sample of 142 informants using a paper-based questionnaire in eight villages. An average number of 17.75 informants were interviewed per village. Informants were averagely aged of 42.92 years old. The majority (52.82%) were young (20 to 40 years) followed by 28.87% of adults (40 to 65 years old) and 18.31% of old (65 to 95 years). Less than half (40.85%) of informants was female against 59.15% of male. We obtained prior informed consent from informants before each interview and this study received the authorization from the University of Parakou Biomedical Research Ethic Committee (CLERB-UP). All the interviews were conducted in accordance with the relevant guidelines and regulations. For each site, we asked informants to list the different utilizations of the species inventoried in both systems (agroforestry systems and surrounding natural habitats). The ethnobotanical value was estimated for each site as the use value index^70^ of species per plot which was computed with the package *ethnobotanyR*^71^ in R. For each species, we asked each informant if the species was used or not for four different types of ethnobotanical uses (food, medicine, religious, construction). Species received 1 if they were used for a specific use category and 0 if not. The use value index for each plant species was calculated following Phillips and Gentry^70^ as the sum of the total number of use citations by all informants for a given species, divided by the total number of informants who cited this species. We calculated the use value index at the plot level as the average use value index of all the species recorded in this plot. This represents the ethnobotanical value of the site. When this value is high, the plot has high number of species that are valuable for various purposes including medicinal, food, spiritual and construction uses.

### Statistical analysis

We performed the Nonmetric Multidimensional Scaling (NMDS) using the *vegan* package^72^ in R version 3.1.2 to assess the degree of dissimilarity in species composition between agroforestry systems and their adjacent natural community. To further understand how these two plant communities are different, we conduct a rank-frequency analysis, plotting species frequency in our survey plots against their ranks. To test the relationship between species richness or Shannon diversity index in agroforestry systems and in adjacent natural habitats, we used linear bootstrap regressions with 1000 iterations using the package *boot*^73^ in R version 3.1.2. Bootstrap regression was also used to test the effect of the type of crops systems, ethnobotanical value and the distance between agroforestry systems and nearby natural habitat on the Shannon diversity index. To test the effects of the three variables on agroforestry systems species richness (count data), we performed a generalized linear model with a negative binomial error structure given the overdispersion of the species richness. We built several candidate models including all single and interactive effects. For each candidate model we calculated the small sample size corrected Akaike Information Criterion, AIC_c_^74^. We then calculated for each model, their ΔAIC_c_ which is calculated as the difference between AIC_c_ of each candidate model and the smallest AIC_c_ value obtained. We selected the best models as the models with ΔAIC_c_ < 2^75^.

In addition, we tested the log–log linear relationship between the proportion of shared species richness and the regional species richness to investigate the influence of regional species pools on local species assemblages in agroforestry systems. We used generalized linear models with a beta error structure to test the effect of the distance from agroforestry system to the adjacent natural habitat, ethnobotanical value, and the type of crops systems on the proportion of shared species richness between agroforestry systems and natural vegetation. We parameterized several candidate models including interactions terms. Similarly to previous models, we used small sample size corrected Akaike Information Criterion^74^ to select the best models.

### Informed consent

This study received the authorization from the University of Parakou Biomedical Research Ethic Committee (CLERB-UP). Prior informed consent was obtained from each participant before the interview.

## Data Availability

The data used for this study are available from the corresponding author on reasonable request.

## References

[1] Swenson NG, Enquist BJ, Pither J, Kerkhoff AJ, Boyle B, Weiser MD, Elser JJ, Fagan WF, Forero-montaña J, Fyllas N, Kraft NJB, Lake JK, Moles AT, Patiño S, Phillips OL, Price CA, Reich PB, Quesada CA, Stegen JC, Monteagudo A (2012). The biogeography and filtering of woody plant functional diversity in North and South America. Glob. Ecol. Biogeogr..

[2] Wallace AR (1878). Tropical Nature and Other Essays.

[3] Connell JH, Slatyer RO (1977). Mechanisms of succession in natural communities and their role in community stability and organization. Am. Nat..

[4] Michalet R, Pugnaire FI (2016). Facilitation in Communities: Underlying Mechanisms, Community and Ecosystem Implications.

[5] Connell JH (1978). Diversity in tropical rain forests and coral reefs. Sci. Am. Nat..

[6] Grime JP (1973). Competitive exclusion in herbaceous vegetation. Nature.

[7] Wilkinson DM (1999). The disturbing history of intermediate disturbance. Oikos.

[8] Al-Mufti MM, Sydes CL, Furness SB, Grime JP, Band SR (1977). A quantitative analysis of shoot phenology and dominance in herbaceous vegetation. J. Ecol..

[9] Huston MA (2014). Disturbance, productivity, and species diversity: Empiricism vs. logic in ecological theory. Ecology.

[10] Silvcrtown J (2004). Plant coexistence and the niche. Trends Ecol. Evol..

[11] Zobel M (1997). The relative of species pools in determining plant species richness: An alternative explanation of species coexistence?. Trends Ecol. Evol..

[12] Cornwell WK, Schwilk DW, Ackerly DD (2006). A trait-based test for habitat filtering: Convex hull volume. Ecology.

[13] Kraft NJ, Valencia R, Ackerly DD (2008). Functional traits and niche-based tree community assembly in an Amazonian forest. Science.

[14] Swenson G, Enquist BJ (2009). Opposing assembly mechanisms in a neotropical dry forest: Implications for phylogenetic and functional community ecology. Ecology.

[15] Cavender-Bares J, Kozak KH, Fine PV, Kembel SW (2009). The merging of community ecology and phylogenetic biology. Ecol. Lett..

[16] Cadotte MW, Tucker CM (2017). Should environmental filtering be abandoned?. Trends Ecol. Evol..

[17] Cariton JT, Geller JB (1993). Ecological roulette: The global transport of nonindigenous marine organisms. Science.

[18] Connolly LMTSR (2013). Understanding diversity–stability relationships: Towards a unified model of portfolio effects. Ecol. Lett..

[19] Mccann KS (2000). The diversity–stability debate. Nature.

[20] Baliddawa CW (1985). Plant species diversity and crop pest control. An analytical review. Int. J. Trop. Insect Sci..

[21] Clara Nicholls MA (2015). Plant biodiversity enhances bees and other insect pollinators in agroecosystems. A review. Agron. Sustain. Dev.

[22] Haddad NM, Crutsinger GM, Gross K, Haarstad J, Tilman D (2011). Plant diversity and the stability of foodwebs. Ecol. Lett..

[23] Guyot V, Castagneyrol B, Vialatte A, Deconchat M (2016). Tree diversity reduces pest damage in mature forests across Europe. Biol. Lett..

[24] Fu H, Yuan G, Jeppesen E, Ge D, Li W, Zou D, Huang Z, Wu A, Liu Q (2019). Local and regional drivers of turnover and nestedness components of species and functional beta diversity in lake macrophyte communities in China. Sci. Total Environ..

[25] MacDougall AS, McCune JL, Eriksson O, Cousins SA, Pärtel M, Firn J, Hierro JL (2018). The Neolithic Plant Invasion Hypothesis: The role of preadaptation and disturbance in grassland invasion. New Phytol..

[26] Mouquet N, Munguia P, Kneitel JM, Miller TE (2003). Community assembly time and the relationship between local and regional species richness. Oikos.

[27] Sferra CO, Hart JL, Howeth JG (2017). Habitat age influences metacommunity assembly and species richness in successional pond ecosystems. Ecosphere.

[28] Macarthur RH, Wilson EO (1963). An equilibrium theory of insular zoogeography. Int. J. Org. Evol..

[29] Simberloff DS (1974). Equilibrium theory of island biogeography and ecology. Annu. Rev. Ecol. Syst..

[30] Vijay, V., & Armsworth, P. R. Pervasive cropland in protected areas highlight trade-offs between conservation and food security. *PNAS* **118**(4), e2010121118 (2021). 10.1073/pnas.2010121118 PMC7848742 33468666

[31] Batisse M (1985). Action plan for biosphere reserves. Environmental conservation.

[32] MAB. Criteria for Designation and Evaluation of Unesco Biosphere Reserves in Germany. German National Committee for the UNESCO Programme (2007).

[33] UNESCO. Biosphere Reserves. The Seville Strategy and the Statutory Framework of the World Network. Paris, France (1995).

[34] Hadush M, Holden ST, Tilahun M (2019). Does population pressure induce farm intensification? Empirical evidence from Tigrai Region, Ethiopia. Agric. Econ..

[35] Tilman D, Fargione J, Wolff B, D’antonio C, Dobson A, Howarth R, Schindler D, Schlesinger WH, Simberloff D, Swackhamer D (2001). Forecasting agriculturally driven global environmental change. Science.

[36] Holden E, Linnerud K, Banister D (2014). Sustainable development: Our common future revisited. Global Environmental Change.

[37] M’Woueni D, Gaoue OG, Balagueman RO, Biaou HS, Natta AK (2019). Road mediated spatio-temporal tree decline in traditional agroforests in an African biosphere reserve. Glob. Ecol. Conserv..

[38] Yaméogo, G., Yélémou, B., & Traoré, D. Pratique et perception paysannes dans la création de parc agroforestier dans le terroir de Vipalogo (Burkina Faso). Base (2005).

[39] Vodouhè FG, Adegbidi A, Coulibaly O, Sinsin B (2011). Parkia biglobosa (Jacq.) R. Br. Ex Benth. Harvesting as a tool for conservation and source of income for local people in Pendjari Biosphere Reserve. Acta Botanica Gallica.

[40] Vodouhè FG, Coulibaly O, Biaou G, Sinsin B (2011). Traditional agroforestry systems and biodiversity conservation in Benin (West Africa). Agrofor. Syst..

[41] Bee JN, Kunstler G, Coomes DA (2007). Resistance and resilience of New Zealand tree species to browsing. J. Ecol..

[42] Hoffmann WA (1996). The effects of fire and cover on seedling establishment in a neotropical savanna. J. Ecol..

[43] Gnangle PC, Egah J, Baco MN, Gbemavo CD, Kakaï RG, Sokpon N (2012). Perceptions locales du changement climatique et mesures d’adaptation dans la gestion des parcs à karité au Nord-Bénin. Int. J. Biol. Chem. Sci..

[44] Ouoba HY, Bastide B, Coulibaly-Lingani P, Kabore SA, Boussim JI (2018). Connaissances et perceptions des producteurs sur la gestion des parcs à Vitellaria paradoxa CF Gaertn. (Karité) au Burkina Faso. Int. J. Biol. Chem. Sci..

[45] Alencar NL, de Sousa Araújo TA, de Amorim ELC, de Albuquerque UP (2010). The inclusion and selection of medicinal plants in traditional pharmacopoeias—Evidence in support of the diversification hypothesis. Econ. Bot..

[46] Gaoue OG, Coe MA, Bond M, Hart G, Seyler BC, McMillen H (2017). Theories and major hypotheses in ethnobotany. Economic Botany.

[47] Helm J, Dutoit T, Saatkamp A, Bucher SF, Leiterer M, Römermann C (2019). Recovery of Mediterranean steppe vegetation after cultivation: Legacy effects on plant composition, soil properties and functional traits. Appl. Veg. Sci..

[48] Nash KL, Graham NA, Jennings S, Wilson SK, Bellwood DR (2016). Herbivore cross-scale redundancy supports response diversity and promotes coral reef resilience. J. Appl. Ecol..

[49] Lambin EF, Turner BL, Geist HJ, Agbola SB, Angelsen A, Bruce JW, Coomes OT, Dirzo R, Fischer G, Folke C (2001). The causes of land-use and land-cover change: Moving beyond the myths. Glob. Environ. Change.

[50] Camou-Guerrero A, Reyes-García V, Martínez-Ramos M, Casas A (2008). Knowledge and use value of plant species in a rarámuri community : A gender perspective for conservation. Hum. Ecol..

[51] de Wet H, Nkwanyana MN, Vuuren VSF (2010). Medicinal plants used for the treatment of diarrhoea in northern Maputaland, KwaZulu-Natal Province, South Africa. J. Ethnopharmacol. J..

[52] Toledo, V. M., Ortiz-Espejel, B., Cortéz, L., Moguel, P., A., & Ordoñez, M. D. J. The multiple use of tropical forests by indigenous peoples in Mexico: A case of adaptive management. *Conserv. Ecol.* **7**(3), 9. https://www.ecologyandsociety.org/vol7/iss3/art9/ (2003).

[53] Azihou FA (2008). Elephants’ (Loxodonta africana) Impacts on Vegetation Structure and Availability of Plant Species that Other Animals Feed on in the Biosphere Reserve of Pendjari.

[54] Faure PVB (1998). Some factors affecting regional differentiation of the soils in the Republic of Benin (West Africa). CATENA.

[55] Tiomoko D (2014). Gestion de la Réserve de Biosphère de la Pendjari : Modes de gestion et proposition d’un modèle conceptuel de durabilité.

[56] ASECNA. Données climatiques, station de Natitingou. Bénin (2010).

[57] PNP. Plan d’Amenagement Participatif et de Gestion du Parc National de la Pendjari, Bénin 2004–2013 (2009).

[58] Assédé EP, Adomou AC, Sinsin B (2012). Magnoliophyta, biosphere reserve of Pendjari, Atacora province, Benin. Check List.

[59] Houinato M, Sinsin B (2000). La pression agro-pastorale sur la zone riveraine de la Réserve de la Biosphère de la Pendjari. Tropicultura.

[60] Gaoue, O. G. Determinant factors for the integrated management of Pendjari hunting reserve northern Benin (Université d'Abomey Calavi, 2000).

[61] Adomou, C. A. Vegetation patterns and environmental gradients in Benin: Implications for biogeography and conservation. PhD. Dissertation, Wageningen University. PhD. Dissertation, Wageningen University (2005).

[62] Inoussa M, Padonou EA, Lykke AM, Glele Kakai R, Bakasso Y, Mahamane A, Saadou M (2017). Contrasting population structures of two keystone woodland species of W National Park, Niger. S. Afr. J. Bot..

[63] Shannon CE (1948). A mathematical theory of communication. Bell Syst. Tech. J..

[64] R Core Team.R: A language and environment for statistical computing. R Foundation for Statistical Computing, Vienna, Austria. https://www.R-project.org/. (2015).

[65] Chao A, Lee S-M (1992). Estimating the number of classes via sample coverage. J. Am. Stat. Assoc..

[66] Bernard HR (2017). Research Methods in Anthropology: Qualitative and Quantitative Approaches.

[67] Ceuterick M, Vandebroek I, Torry B, Pieroni A (2008). Cross-cultural adaptation in urban ethnobotany: The Colombian folk pharmacopoeia in London. J. Ethnopharmacol..

[68] Cohen N, Arieli T (2011). Field research in conflict environments: Methodological challenges and snowball sampling. J. Peace Res..

[69] Tongco MDC (2007). Purposive sampling as a tool for informant selection. Ethnobot. Res. Appl..

[70] Phillips O, Gentry AH (1993). The useful plants of Tambopata, Peru: I. Statistical hypotheses tests with a new quantitative technique. Econ. Bot..

[71] Whitney, C. EthnobotanyR: Calculate Quantitative Ethnobotany Indices. Package Version 0.1.8 https://CRAN.R-project.org/package=ethnobotanyR (2021).

[72] Oksanen M, Lehtonen S, Jaronen M, Goldsteins G, Hämäläinen RH, Koistinaho J (2019). Astrocyte alterations in neurodegenerative pathologies and their modeling in human induced pluripotent stem cell platforms. Cell. Mol. Life Sci..

[73] Canty, A. J. Resampling methods in R: The boot package. R News **2**(3): 2–7 (2002).

[74] Akaike H (1973). Maximum likelihood identification of Gaussian autoregressive moving average models. Biometrika.

[75] Burnham KP, Anderson DR (2004). Multimodel inference: Understanding AIC and BIC in model selection. Sociol. Methods Res..

